# Invalid measles vaccine dose administration and vaccine effectiveness in Ethiopia

**DOI:** 10.11604/pamj.2021.40.229.29028

**Published:** 2021-12-16

**Authors:** Teklay Desta, Ephrem Lemango, Daddi Wayessa, Liya Wondowossen, Mirkuzie Kerie, Balcha Masresha

**Affiliations:** 1 Maternal and Child Health Directorate, FMoH Ethiopia, P.O. Box 1234, Addis Ababa, Ethiopia,; 2 Ethiopian Public Health Institute, FMoH. P.O. Box 1242, Addis Ababa, Ethiopia,; 3 World Health Organization, Regional Office for Africa, Brazzaville, Congo

**Keywords:** Measles elimination, measles invalid doses, vaccine effectiveness, Ethiopia

## Abstract

**Introduction:**

Ethiopia endorsed the African regional measles elimination goal in 2012 and has been implementing measles elimination strategies. Administration of measles vaccine before the age of nine months decreases seroconversion. Ensuring administration of valid doses and monitoring vaccine effectiveness is crucial for achieving measles elimination. The objective of the study was to describe the magnitude of invalid measles dose administration and vaccine effectiveness in Ethiopia.

**Methods:**

we analysed the 2016 Ethiopian Demographic and Health Survey (EDHS) immunization coverage data for Ethiopia to determine the age at measles vaccine administration and proportion of measles age invalid doses administered. The national measles surveillance data for children with birthdates that match 12-23 months old children surveyed in the EDHS 2016, were analysed to determine the Proportion of Cases Vaccinated (PCV) with one dose of measles vaccine. We estimated the effectiveness of measles vaccine by using the proportion of measles cases vaccinated (PCV) from measles surveillance data and the measles vaccination coverage among children aged 12-23 months reported in the demographic health survey (DHS) done in 2016 (Percent of Population Vaccinated for measles, PPV). The screening method was used to estimate measles vaccine effectiveness at national level and for regions which reported more than 30 measles cases among children 9-23 months of age in the 2013-2015 period. The correlation between the median age of invalid doses administered, proportion of invalid doses and measles vaccine effectiveness was analysed.

**Results:**

at national level, the proportion of invalid measles dose administration was 27.6% for children aged 12-35 months surveyed in the 2016 DHS survey in Ethiopia. Among children reported in the measles case-based surveillance database with birthdates that match the children surveyed in the Ethiopian DHS 2016, the proportion of measles cases vaccinated with a single dose of measles vaccine in the 2013-2015 period was 22.7%. The vaccine effectiveness for single dose measles vaccination was estimated at 75.3%. The measles vaccine effectiveness was low for regions with high proportion of invalid dose administration and lower median age of invalid dose administration. The median age of measles dose administered before the age of nine months was significantly correlated with measles vaccine effectiveness (r=0.971, p=0.001) in the respective regions.

**Conclusion:**

the proportion of invalid measles dose administration is very high in Ethiopia and is associated with lower vaccine effectiveness. Further assessment should be carried out to understand the underlying root causes for invalid dose administration, focusing on areas with high proportion of invalid measles doses. The national program should devise strategies to promote timely vaccination as per the national schedule, and to revaccinate those vaccinated before 9 months of age. The ministry of health should also strengthen the platform for immunization in the 2^nd^ year life, to ensure high routine immunization coverage with two doses of measles vaccine to achieve the measles elimination goal in Ethiopia.

## Introduction

Measles is one of the leading causes of death among young children globally. Accelerated immunization activities have had a major impact on reducing measles deaths. During 2000-2017 measles vaccination has prevented an estimated 21.1 million deaths globally, and measles deaths have decreased by 80% from an estimated 545,174 in 2000 to 109,638 in 2017 [1]. Globally, it is estimated that 20.8 million infants did not receive measles vaccine through routine immunization in 2017. Ethiopia is one of the 6 countries with the biggest number of infants who missed measles vaccination in 2017 [2].

Ethiopia has been implementing the Reaching Every District approach since the year 2004 as the recommended technical approach to improve routine immunization coverage. The first dose of measles vaccine (MCV1) is provided at 9 months of age or shortly thereafter in the national immunization schedule. The administrative MCV1 coverage for Ethiopia increased from 65% in 2007 to 93% in 2016 [3-5]. However, EPI cluster coverage survey conducted in 2012 and Demographic and Health Survey (DHS) conducted in 2016 showed measles vaccination coverage of 68.2% and 54.3% respectively [6, 7]. On the other hand, the WHO/UNICEF Estimates of National Immunization Coverage (WUENIC) give MCV1 coverage of 59%, 55% and 58% for 2017, 2018 and 2019 respectively [8]. In February 2019, Ethiopia introduced the second dose of Measles Containing Vaccine (MCV2) in the routine immunization schedule, providing it at 15 months of age.

Ethiopia started implementing the measles mortality reduction strategies in 2002, with the first nationwide measles catch-up Supplemental Immunization Activities (SIAs) conducted in 2002-2003 and several follow up SIAs conducted between 2003 and 2017 [9, 10]. In 2011, Ethiopia endorsed the African regional measles elimination goal for 2020 [10] and developed a national measles elimination plan in 2012 [11]. The key strategies recommended for accelerating the control of measles include strengthening routine immunization, supplemental immunization, enhanced case-based measles surveillance, and management of measles cases including vitamin A supplementation [4].

Measles case-based surveillance has been in place in Ethiopia since 2004, supplemented by laboratory confirmation [12]. The non-measles febrile rash illness rate and the proportion of districts investigating at least one suspected case of measles with a blood specimen were 1.8 per 100,000 population and 95% respectively in 2006 [9]. In 2015, the non-measles febrile rash illness rate was 2.3 per 100,000 population, while 76% of districts investigated at least one suspected case of measles [13].

Passive maternal antibodies are known to provide protection against measles in the first months of life. The proportion of infants who seroconvert depends on the age of MCV1 vaccination. The seroconversion to measles vaccine provided at 9 months of age is estimated to be around 85% [14, 15]. In a meta-analysis of 20 studies conducted on measles vaccination given before the age of 9 months, measles seroconversion increased from 50% (95%; CI 29-71%) at 4 months of age to 67%(95% CI 51-81%) at 5 months,76% (95% CI 71-82%) at 6 months, 72%(95% CI 56-87%) at 7 months and 85% (95 CI 69-97%) at 8 months [16]. In terms of the duration of immunity, there is significantly faster measles antibody waning with measles vaccination before 9 months of age as compared to vaccination at or after 9 months of age [16].

Measles vaccine doses administered before the age of nine months are considered invalid doses [17]. Measles vaccine doses may be provided to children starting at 6 months of age in outbreak settings in an effort to provide protection to young infants. However, this does not constitute the regular first dose and should not be documented as such, and these children are expected to receive their MCV1 dose at 9 months of age [18]. Different datasets and studies conducted in Ethiopia have shown that high proportion of measles vaccine is administered before the age of nine months. An immunization coverage survey conducted in Tselemti district of Ethiopia in 2000 reported that 25.4% of the children surveyed were vaccinated for measles before the age of nine months [19]. The 2006 national immunization coverage survey reported that only 78.8% of measles vaccine doses were reported to be valid according to the national immunization schedule for measles [20]. An investigation of recurrent measles outbreaks in SNNPR in 2014 found that an estimated 20% of children received measles vaccine before 9 months of age [21]. From DHS 2011 analysis, the proportion of children vaccinated before 9 months age in routine immunization in Ethiopia was reported to be 26.3% [22, 23].

Vaccine effectiveness analysis serves as a critical method of evaluation of the immunization program and vaccination coverage [24]. Measles vaccine effectiveness at population level can be estimated from measles case-based surveillance reports [24-26]. This manuscript describes the magnitude and distribution of invalid measles dose administration and attempts to estimate vaccine effectiveness in Ethiopia using data from the measles surveillance database and the Ethiopian DHS 2016.

## Methods

We analysed the measles case-based surveillance data for the years 2013-2015 and the Ethiopian Demographic and Health Survey (EDHS) 2016 data.

**Measles case-based surveillance data:** in Ethiopia, measles is an immediately reportable disease. In this case-based surveillance system, each suspected measles case is investigated and reported using a standard case reporting form, and a blood specimen is collected to test for measles immunoglobulin M (IgM). The measles case-based surveillance data includes standard epidemiological variables like the unique identification number, Region/Zone/district of residence, age, date of onset of rash, the number of measles vaccine doses received, IgM test results, final classification of cases and the final outcome of the case.

**Ethiopian Demographic and Health Survey (EDHS) 2016 data:** the 2016 Ethiopian Demographic and Health Survey is the fourth DHS conducted in Ethiopia. It is implemented by the Central Statistics Agency (CSA) at the request of the Federal Ministry of Health (FMoH). Data collection took place from 18^th^January 2016 to 27^th^ June 2016. The Ethiopian DHS collected data on key aspects of family health, including immunization coverage. EDHS data include variables like the Region, birthdate, measles vaccine receipt date (retrieved from the child immunization card or from records in health facility where the child received immunization [7].

**Data analysis:** we reviewed the measles case-based surveillance database and analysed the vaccination status of measles cases aged 9-23 months old children reported in the 1 January 2013 to 31^st^ December 2015 period and calculated Proportion of Cases Vaccinated (PCV) with one dose of measles vaccine. Children with births dates January 18^th^, 2013 to June 27^th^, 2015 who were reported as confirmed measles case aged 9-23 months were included in the analysis. Children who were reported to receive more than one dose of measles vaccine were excluded from the analysis. The children born between January 18^th^, 2013 to June 27^th^, 2015 match with the births dates of children surveyed in the EDHS 2016.

The Ethiopian DHS data were analysed to calculate the proportion of age invalid measles doses administered before the age of 9 months for 12-35 months old, surveyed children. The measles coverage (Percent of Population Vaccinated, PPV) for children aged 12-23 months old reported in the Ethiopian DHS was used to calculate measles vaccine effectiveness.

The measles surveillance data was recoded to create categorical variables. Laboratory confirmed, clinically suspected or compatible, and epidemiologically linked suspected measles cases were classified as confirmed measles cases as defined in the national measles surveillance and outbreak management guideline for measles surveillance and outbreak management [12]. Measles cases reported to have received one dose of measles vaccine in the database were classified as “vaccinated with one dose of measles vaccine”, while measles cases with zero doses and unknown status were classified as “not vaccinated”. The birthdates of children in the surveillance data were calculated by deducting the age of children in days from the onset of measles disease.

The equation developed by Orenstein and colleagues in 1980 that was developed to determine the Proportion of Cases Vaccinated (PCV) in the field during an outbreak was used to drive the equation to determine vaccine effectiveness [24, 26].


$$PCV=\frac{PPV-(PPV*\;VE)}{1-(PPV*VE)}$$

Where PCV is the proportion of cases vaccinated; PPV is the proportion of the population vaccinated. The following equation was derived from the PCV formula to estimate Vaccine Effectiveness (VE). This screening method is a simple, rapid and economical way of estimating vaccine effectiveness [27, 28].


$$VE=\frac{PCV-PPV}{PPV*(PCV-1)}$$

Age at measles vaccine receipt date was calculated from the DHS data by subtracting childbirth date from the measles vaccine receipt date. The median age of the invalid measles doses administered for children aged 12-35 months was analysed by region. The proportion of invalid doses was calculated for children who had measles receipt date recorded. The proportion of invalid doses for those vaccinated before six months of age and before nine months of age was calculated by region and at the national level. The correlation between the median age of invalid doses administered, proportion of invalid doses and measles vaccine effectiveness was analysed.

### Operational definitions

**Measles age invalid doses:** measles vaccine doses administered before the age of nine months (less than 270 days). The age in days at receipt of measles vaccine was calculated by subtracting the Century Day Code (CDC) of birth from the Century Day Code (CDC) of measles vaccine receipt for children with documented measles vaccine receipt dates in the EDHS data.

**Measles doses administered too early:** measles doses that were administered before the age of six months or 180 days of age.

**Proportion of invalid doses:** number of measles doses administered before nine months (or less than 270 days) divided by total number of children with vaccine receipt dates.

**Vaccinated with one dose of measles vaccine:** is defined as a measles case reported in the measles case-based surveillance that was reported to be vaccinated with one dose of measles vaccine before the onset of measles disease.

**Not vaccinated:** measles cases reported in the measles case-based surveillance reported to have not received any measles vaccine or are reported with unknown measles vaccination status.

**Proportion of cases vaccinated (PCV):** with one dose of measles vaccine is defined as the number of measles cases that were reported to be vaccinated with one dose of measles vaccine among all the reported measles cases during the same period.

**Percent of the population vaccinated (PPV):** the measles coverage of the population - the measles routine vaccination coverage among children aged 12-23 months old reported in the EDHS 2016 survey.

**Vaccine effectiveness (VE):** is the percentage reduction of disease incidence in a vaccinated group compared with an unvaccinated group in the field in the real world under non-ideal conditions and in different populations [26].

## Results

**Distribution of DHS surveyed children, and measles cases from surveillance data:** the 2016 Ethiopian DHS sampled a total of 3855 children aged 12-35 months (1929 children aged 12-23 and 1926 children aged 24-35 months). Date of vaccination was available for only 1427children (774 children aged 12-23 months and 653 children aged 24-35 months), while 1635 were not vaccinated and the mothers/caretakers did not know if the child got measles vaccine in 40 of the children. Out of those vaccinated for measles, 724 of them were reported to be vaccinated by maternal recall, and in another 29 there was written proof of vaccination for measles in their child immunization card with no date. Out of those 1427 with measles vaccine receipt date, 344 were vaccinated before the age of 9 months, 234 were vaccinated after the age of one year and 849 were vaccinated between 9-12 months of age. The median age of the invalid measles doses administered for children aged 12-35 months was analysed by region. The median age among those vaccinated before the age of nine months was 258 days in Addis Ababa, while it was 238 days in Oromia region (Table 1).

**Table 1:** age at measles vaccination for children 12-35 months old children, Ethiopian DHS 2016

Region	*Age at measles vaccination for children 12-35 month old surveyed in Ethiopian DHS2016.					**Median age (months) of measles vaccination for those vaccinated before 9 months age
	< 9 months	9-11 months	>=12 months	missing (no vaccination receipt date)	Total	
Addis Ababa	13	149	4	19	185	258
Afar	4	20	11	350	385	215.5
Amhara	37	80	22	205	344	254
B/Gumuz	39	71	40	162	312	254
Dire Dawa	19	89	27	69	204	242
Gambella	13	35	13	197	258	256
Harari	16	64	10	135	225	241.5
Oromia	28	55	25	462	570	238
SNNPR	78	83	38	256	455	244.5
Somali	25	35	17	435	512	247
Tigray	72	168	27	138	405	256
Grand Total	344	849	234	2428	3855	251

* Age at measles vaccination for children aged 12-35 month old during the Ethiopian DHS, for those vaccinated for measles and with vaccination date recorded. **Median age of measles vaccination for those vaccinated before 9 months age (among children aged 12-35 surveyed during the DHS 2016)

The measles case-based surveillance database contains a total of 37,585 cases of confirmed measles cases reported between January 1^st^ 2013 to December 31^st^ 2015, of which, 6539, 13,303 and 17,743 were reported in 2013,2014 and 2015 respectively. Over 91% of the cases were reported from the regions of Oromia (54%), Amhara (17%) and SNNPR (20%). Only 40% of the cases were under five years old (<60 months) children (Table 2).

**Table 2:** confirmed measles cases among children with birth dates between 18^th^ January 2013 and 27^th^ January 2015 reported in the 2013-2015 case-based measles surveillance system by age group and vaccination status, Ethiopia

Region	Age group of confirmed measles cases				Measles vaccination status of 9-23 months old, confirmed cases			
	0-8 months	9-23 months	>23 months	Total confirmed cases	Not vaccinated (0 dose)	Vaccinated (1 dose)	Total	% of vaccinated with one dose of measles vaccine
Addis Ababa	69	50	498	617	4	28	32	12.5%
Afar	5	39	206	250	0	27	27	0.0%
Amhara	151	521	5905	6577	79	345	424	18.6%
B/Gumuz	20	14	227	261	0	10	10	0.0%
Dire Dawa	1	10	47	58	0	5	5	0.0%
Gambella	62	113	435	610	14	74	88	15.9%
Harari	1	12	34	47	1	4	5	20.0%
Oromia	1110	2704	16590	20404	562	1768	2330	24.1%
SNNPR	489	634	6352	7475	80	248	328	24.4%
Somali	97	26	460	583	1	24	25	4.0%
Tigray	48	59	596	703	11	25	36	30.6%
Total	2053	4182	31350	37585	752	2558	3310	22.7%

**Invalid dose administration:** the weighted national proportion of invalid doses (measles doses received before nine months of age) was 27.6% (26.8% among the 24-35 months old and 28.5% for those aged 12-23 months respectively). The proportion of invalid doses varied significantly among regions. Addis Ababa city administration had the lowest proportion of invalid doses 7.8%, on the other hand, Southern Nations, Nationalities, and Peoples' Region (SNNPR) had the highest proportion of invalid doses 39.2% for 12-35 months old children. Similarly, the proportion of children vaccinated too early (before six months of age) was calculated for the 12-35 months old children. Addis Ababa city administration had the lowest (0.6%) and Oromia region had the highest (8.3%) invalid doses administered before the age of six months (Table 3).

**Table 3:** proportion of children vaccinated before 6 months of age and before 9 months of age among 12-35 months old children, DHS 2016, Ethiopia

Region	Timing of receipt of measles vaccination among 12-35 months old children with known vaccine receipt date (DHS2016)				
	# with recorded vaccine receipt date	vaccinated too early (before 6 months of age)	vaccinated early (before 9 months of age)	% vaccinated too early (before 6 months of age)	% vaccinated early (before 9 months of age)
Addis Ababa	166	1	13	0.6%	7.8%
Afar	35	1	4	2.9%	11.4%
Amhara	139	5	37	3.6%	26.6%
B/Gumuz	150	2	39	1.3%	26.0%
Dire Dawa	135	3	19	2.2%	14.1%
Gambella	61	0	13	0.0%	21.3%
Harari	90	3	16	3.3%	17.8%
Oromia	108	9	28	8.3%	25.9%
SNNPR	199	10	78	5.0%	39.2%
Somali	77	3	25	3.9%	32.5%
Tigray	267	7	72	2.6%	27.0%
Total	1427	44	344	5.2%	27.6%

**Effectiveness of measles vaccine:** there were 37,585 confirmed measles cases reported between 1^st^ January 2013 and 31^st^ December 2015, out of which 3,310 were children born between 18^th^ January 2013 and 27^th^ June 2015 and reported as measles cases at the age of 9-23 months. Among the 3,310 confirmed measles cases aged 9-23 months, it was found that 752 cases (22.7%) were vaccinated with single dose of measles vaccine, 2,558 cases were not vaccinated at all or had unknown vaccination status. The proportion of measles cases vaccinated with single dose of measles vaccine was analysed for six regions (Addis Ababa, Amhara, Gambella, Oromia, SNNPR and Tigray) which had more than 30 confirmed measles cases in the 2013-2015 period reported in the 9-23 months age group (Table 2).

**Figure 1:**
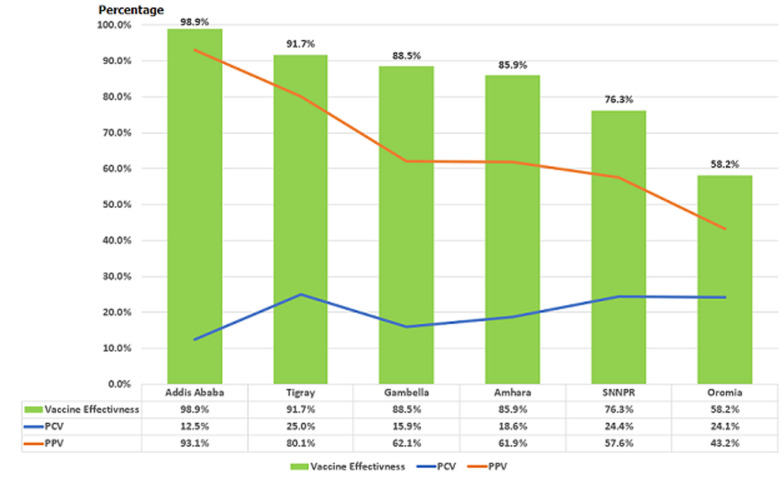
proportion of cases vaccinated (PCV) with one dose of measles vaccine, percent of population vaccinated (PPV) and measles vaccine effectiveness (VE) for 9-23 months old children reported in measles case based surveillance 1^st^ January 2013 to 31^st^ December 2015

Nationally, the effectiveness of one dose of measles vaccine was estimated at 75.3% using the EDHS 2016 reported 54.3% measles coverage (Percent of Population Vaccinated, PPV) for 12-23 months old children surveyed in 2016 and 22.7% proportion of measles cases vaccinated for the children from measles case surveillance data with birthdates that match those surveyed in the EDHS 2016. The measles vaccine effectiveness was highest for Addis Ababa (98.9.%) followed by Tigray (91.7%), Gambella (88.5%) and Amhara Regions (85.9%). On the other hand, the measles vaccine effectiveness was 58.2% in Oromia and 76.3% in SNNPR. The proportion of cases vaccinated (PCV) for measles, the Percent of Population Vaccinated (PPV) for measles, which is the measles coverage reported in EDHS 2016 for children 12-23 months old in the survey and the estimated Vaccine Effectiveness (VE) for one dose of measles for the six regions is depicted in (Figure 1). Regions with low coverage (PPV) and high PCV have low vaccine effectiveness.

**Figure 2:**
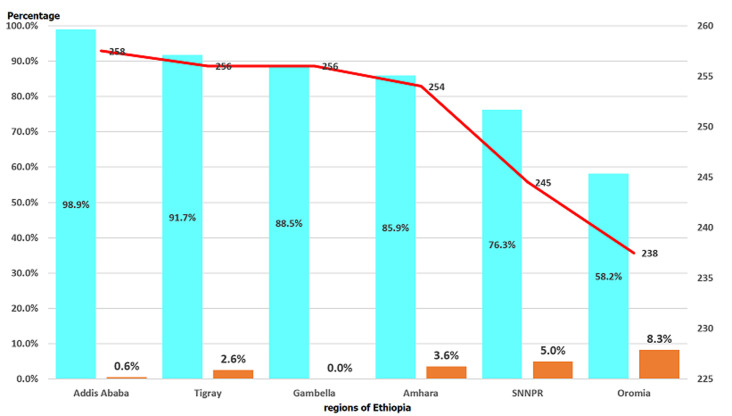
median age of measles cases (in days of age) in children who received measles vaccine before the age of 9 months, proportion of invalid measles dose administered before 6 months (180 days) of age and estimated measles effectiveness in six regions of Ethiopia, 2013-2015

Measles vaccine effectiveness was significantly and negatively correlated with the proportion of measles doses administered before the age of six months (180 days) in the six regions (r=-0.968, p=0.0015). The median age of measles cases who received the vaccine before the age of nine months was also significantly correlated with the measles vaccine effectiveness for the same period (r=0.974, p=0.0001) (Figure 2).

## Discussion

In this study, we analysed the proportion of invalid measles doses administered from the Ethiopian DHS2016 and calculated the proportion of measles cases vaccinated with one dose of measles vaccine from the case-based measles surveillance data to estimate measles vaccine effectiveness nationally and in six regions to examine the impact of invalid doses administration on vaccine effectiveness.

Nationally, measles vaccine effectiveness was estimated at 75.3%, with significant variability ranging from 98.9% in Addis Ababa to 58.2% in Oromia region. Many studies have indicated that measles vaccine effectiveness for single dose of measles vaccine to be at least 85% when administered at 9 months of age [29, 30].

A review of 70 papers published from 1964 to 2010 on measles vaccine effectiveness showed that, for a single dose measles vaccine administered at 9-11 months, the median vaccine effectiveness (VE) was 77.0% (Inter Quartile Range [IQR] 62-91%), and when analysis was restricted to include only point estimates for which vaccination history was verified and cases were laboratory confirmed, the median VE was 84.0% (IQR, 72%-95%) [31]. The estimated national measles VE in our study is within the range of measles VE at 9-11 months referred above. The measles VE reported in this study was also similar with a study from Kerala, India that reported measles VE of 76.6% using the outbreak method [32].

Measles vaccine effectiveness was assessed from field evaluation of measles attack rate among measles cases aged 9-36 months old following an outbreak in Gode Zone of Somali region, Ethiopia in 2000 which shows measles effectiveness of 66.9%, and vaccine administration or cold chain problem was indicated as possible causes for low vaccine effectiveness [33]. According to the DHS 2016 data analysis, 27.6% of children aged 12-35 months were vaccinated against measles before the age of nine months. The measles invalid dose administration in Ethiopia is very high compared to the finding from a study that analysed DHS data for 31 African countries that reported the median proportion of invalid MCV1 administration to be 15.5% (IQR, 10.0-18.1) for the African countries. Ethiopia with 26.3% invalid measles dose administration from DHS 2011 was among the top three countries with high proportion of age-invalid measles doses [20]. This shows that the administration of age invalid measles doses has remained very high in Ethiopia during the years between DHS 2011 and DHS 2016.

The proportion of measles doses administered too early (before 6 months of age) ranged between 8.3% for Oromia region and 0.6% for Addis Ababa city administration. We also found that among the children who received invalid measles doses, the median age of administration was 258 days in Addis Ababa city administration while it was 238 days of age in Oromia region. Too early measles administration was highly associated with lower vaccine effectiveness. Our analysis showed that the vaccine effectiveness was highly correlated with the proportion of too early administered measles doses (r=-0.935, p<0.01) and lower median age of measles invalid dose administration (r=0.971, p<.01). This is consistent with the findings of a meta-analysis of 20 studies conducted on measles vaccination given before the age of 9 months [16]. Hence, the low measles VE in Oromia region can be explained by the lowest median age (238 days) of invalid measles doses administration and highest proportion of too early measles doses administration (among the six regions with more than 30 cases).

**Limitations:** our study has some limitations. The screening method for estimating vaccine effectiveness may lead to an over-estimation of the effectiveness if the proportion of vaccinated persons among the measles cases is overestimated. Vaccination coverage levels are not homogenous within large geographic regions, more so in big regions like Oromia, Amhara and SNNPR in Ethiopia. Therefore, the interpretation of measles vaccine effectiveness has to take into account that district level variation may lead to an increase or a decrease in the estimated vaccine effectiveness. In addition, in the measles case-based surveillance database we used for our study, the vaccination status of measles cases was documented mostly through parental recall as a source of information when the child is first detected with a febrile rash. Therefore, recall bias may lead to an underestimation or overestimation of the measles vaccine effectiveness.

## Conclusion

In conclusion, the proportion of invalid measles doses administration in Ethiopia is very high and is associated with lower vaccine effectiveness. However, the underlying reasons for invalid measles dose administration remain unknown. We strongly recommend the national immunization program to undertake studies to identify reasons why health workers administer measles vaccine before 9 months of age. The factors that led to untimely delivery of measles vaccine may also lead to wrong timing or interval of delivery of other antigens and booster doses. The assessments should be carried out focusing on the districts (or the equivalent) with high proportion of invalid measles doses and devise strategies to ensure timely delivery of measles vaccine doses, as well as strategies to revaccinate those vaccinated before 9 months of age. Health workers should be properly trained and supported through supervision and program reviews to adhere to the right age for vaccine administration. The opportunity created by the introduction of routine immunization doses in the second year of life should be used to build capacity and reinforce monitoring in order to ensure timely vaccination of eligible children, and also help to catch up on the delivery of missed doses, even when the child is beyond 1 year of age. Ethiopia will only be able to make progress towards the measles elimination targets if sustained high coverage is achieved for the first and second doses of measles vaccine, and if the doses are provided at the right age to ensure adequate protection.

### What is known about this topic

- Measles vaccine is a safe and potent with an estimated seroconversion of 85% when the vaccine is administered at 9 months of age;
- Different datasets and studies conducted in Ethiopia have shown that high proportion of measles vaccine is administered before the age of nine months.

### What this study adds

- Measles case-based surveillance and demographic and health survey data combined to estimate measles vaccine effectiveness;
- National and subnational measles vaccine effectiveness estimated. The study will raise the awareness of immunization managers on the magnitude of age invalid measles administration and will motivate regions with reported low vaccine effectiveness and high proportion of age invalid doses to investigate the reasons behind the high proportion of age invalid doses and ensure health workers adhere to national immunization schedule;
- A correlation between the median age of invalid doses administered and vaccine effectiveness demonstrated.
